# Difference in the risk of discrimination on psychological distress experienced by early wave infected and late wave infected COVID-19 survivors in Japan

**DOI:** 10.1038/s41598-023-40345-9

**Published:** 2023-08-12

**Authors:** Megumi Hazumi, Mayumi Kataoka, Kentaro Usuda, Zui Narita, Emi Okazaki, Daisuke Nishi

**Affiliations:** 1 Department of Public Mental Health Research, National Institute of Mental Health, National Center of Neurology and Psychiatry, 4-1-1 Ogawahigashicho, Kodaira, Tokyo 187-8553 Japan; 2 Department of Sleep-Wake Disorder, National Institute of Mental Health, National Center of Neurology and Psychiatry, 4-1-1 Ogawahigashicho, Kodaira, Tokyo 187-8553 Japan; 3 Department of Mental Health, Graduate School of Medicine, The University of Tokyo, 7-3-1 Hongo, Bunkyo-ku, Tokyo, 113-0033 Japan; 4 Department of Behavioral Medicine, National Institute of Mental Health, National Center of Neurology and Psychiatry, 4-1-1 Ogawahigashicho, Kodaira, Tokyo 187-8553 Japan

**Keywords:** Human behaviour, Public health

## Abstract

The psychological distress experienced by coronavirus disease of 2019 (COVID-19) survivors after recovery from the illness is amplified by discrimination endured because of their infection status. However, the difference in the risk of facing discrimination and risk of experiencing psychological distress in the early and late waves of the COVID-19 pandemic remain unclear. This study aimed to investigate whether the risk of facing discrimination because of infection status was lower in the early or late waves and whether risk of discrimination on psychological distress was more serious in later, rather than earlier waves. We conducted two online surveys to collect data from survivors divided into two groups. The participants with scores of five or more on the Kessler Psychological Distress Scale were identified as having experienced psychological distress. The participants were identified as having experienced discrimination based on infection status if they had endured being blamed, some type of discrimination, or having themselves or their families maligned. The timing of infection was split into infected during early waves of the pandemic for 2021 participants and infected during later waves of the pandemic for 2022 participants. Modified Poisson regression analyses were performed using experiences of discrimination as criteria and timing of infection as predictors. Modified Poisson regression analyses were further performed using the presence of psychological distress as a criteria and experiences of discrimination and timing of infection as the criteria, in addition to interaction effect of these es. The data of 6010 participants who were infected in early waves and 5344 participants who were infected in later waves were analyzed. The risks of being blamed, some forms of discrimination, and participants and their families being maligned were significantly lower in the group who were infected in later waves than those infected in earlier waves. Experiences of discrimination were highly associated with psychological distress in those infected in later waves than those infected in earlier waves, while only being blamed showed a significant association. Risk of discrimination was found to be lower in those infected in later waves, whereas risk of discrimination on psychological distress was shown to be more serious in those infected in later waves. Therefore, we submit that it is more important to support COVID-19 survivors who face discrimination, than it is to attempt to decrease the current discriminatory climate caused by the COVID-19 pandemic.

## Introduction

Psychological distress, which includes various psychiatric symptoms, such as post-acute sequelae of COVID-19 (PASC), is still problematic in COVID-19 survivors. PASC has been observed in approximately 10 to 20% of COVID-19 survivors after recovery^1^. One of the PASC of COVID-19 is psychiatric symptoms^2^, with 23% of COVID-19 survivors presenting with anxiety and 12% presenting with depression^3^. One study of Japanese COVID-19 survivors reported that 36% of the participants suffered from psychological distress after discharge^4^. This psychological distress often lasts over a year, with approximately 19.7% of survivors suffering for more than 1 year^5,6^. It has been suggested that such symptoms do not diminish spontaneously over time based on several studies. The study of COVID-19 survivors indicating the prevalence of having depression or anxiety being higher at over 6 months than less than 6 months^3^. Longitudinal studies of the general population during the pandemic also show that depression, anxiety and distress tend to persist or worsen over time^7,8^. Some studies report that the prevalence of psychological distress is similar in those infected in the early and late waves of the COVID-19 pandemic^9,10^. A further study found that among Japanese COVID-19 survivors, psychological distress was more prevalent in those infected in the later waves, than those infected in earlier waves^4^. Considering that psychological distress does not improve spontaneously and that the number of individuals suffering does not decrease, factors associated with symptoms should be identified to attempt to protect survivors from this distress or to improve the severity of symptoms.

Discrimination is often experienced by those individuals who are or have been infected with COVID-19 and this may increase the psychological distress experienced by COVID-19 survivors. A systematic review revealed that 38% of COVID-19 survivors perceived some type of stigma, including discrimination. Some studies conducted in Asian countries have identified that COVID-19 survivors who perceived stigma, including discrimination, were more likely to experience psychological stress^9^. This included 51.1% of the participants in a Chinese study^11^, and 43.3% of participants in a Japanese study^12^. A systematic review of the general population indicates that discrimination affects psychological distress, especially among disadvantaged groups^13^. Such associations between discrimination and psychological distress has been observed in several Asian general populations^14,15^. A recent longitudinal study demonstrated that experiencing discrimination was associated with psychological distress among Asian people during the COVID-19 pandemic^16^. A similar tendency has been observed in COVID-19 survivors. Some studies using cross-sectional surveys have also revealed an association between perceived stigma, including discrimination, and psychological distress^4,17,18^.

Discrimination is suggested to be less frequently experienced by those who were infected in the later waves than those infected in the early waves, while previous studies accounting for the difference have not provided sufficient explanation for this finding. The amount of public knowledge about COVID-19 negatively associates with the degree of stigma including discrimination toward COVID-19^19,20^. Knowledge relating to COVID-19 increases gradually among the general population as time passes^21^. Considering this, the perceived stigma, including discrimination, endured by COVID-19 survivors could diminish as time passes. To our knowledge, only two studies have compared the difference in the discrimination suffered by survivors based on the two time periods, and these results were controversial. One study wherein the difference was compared by month reported that no decrease was found^22^. The other study compared the difference across a period of approximately 8 months and found that discrimination was less prevalent in the later waves than in the earlier waves^10^. Considering these results, it is possible that a longer interval between the comparison of the differences might be associated with less discrimination, but this is not clear since supporting studies have not been carried out. As even more time has elapsed since the outbreak of the virus, we submit that additional comparisons can be made at extended intervals. We believe that further investigation with extra intervals should be performed.

Furthermore, if the risk of being discriminated against is based on infection status and this risk has become less prevalent since the earlier waves of COVID-19, then this should indicate that risk of discrimination impacting psychological distress should be more serious in the later waves than in early wave. This proposition is based on the concept of the group identification^23^ and the rejection-identification model^24^: individuals who experience the similar difficulties tend to form groups and recognizing themselves as a member of a group having difficulty, called as the group identification, which has the role of buffering the psychological distress caused by discrimination^24–26^. Developing group identification is difficult for those who have been discriminated against under the society with a low discriminatory climate^27^, resulting in being forced to endure the psychological distress caused by this discrimination alone. This mechanism may possibly be applied to COVID-19 survivors. Given that the discriminatory climate is lower in the late wave than in the early wave^10,21^, those in the late wave are suspected to be more difficult in dealing with psychological distress caused by discrimination because they have less group identification, but this has not yet been thoroughly investigated. On the other hand, considering many individuals experienced discrimination in the early waves, resources for supporting them might be established and buffer the psychological distress caused by discrimination in the late wave. However, no studies compare the strength of the impact of discrimination on psychological distress between those in the early and late waves to our knowledge. Thus, it is necessary to examine whether the impact of discrimination on psychological distress was stronger or not in the late wave than in the early wave.

Therefore, this study aimed to test the following hypotheses:

1. the risk of experiencing discrimination based on COVID-19 infection status is lower in the later COVID waves than it was in the earlier wave, and
2. the risk of discrimination having a negative effect on psychological distress is more serious in the later waves of COVID than in the earlier waves.

The results of our study may provide suggestions for strategies for dealing with the discrimination which many COVID-19 survivors face and for reducing psychological distress as PASC.

## Methods

### Study design and participants

This was a cross-sectional study and the participants were COVID-19 survivors who were recruited online and asked to complete an online survey. This process was administrated by Rakuten Insight, a large consumer market research data collection company in Japan (Rakuten Insight, Tokyo, Japan). The group of COVID-19 survivors who were infected in the early waves were recruited from July to September 2021 and those who were infected in the later waves were recruited in September 2022. The inclusion criteria were as follows: participants had to (1) be over 20 years old, (2) agree to voluntarily participate, and (3) those who have tested positive on Polymerase Chain Reaction (PCR) tests for COVID-19 in this study. Further inclusion criteria were based on whether the participants had been infected in the early or later waves of the COVID-19 pandemic.

The COVID-19 survivors who were infected in the early waves were identified as those who answered “yes” to the screening question: “Have you ever tested positive on a PCR test for COVID-19?”. The COVID-19 survivors who were infected in the later waves were identified as those who answered “yes” to the question: “Have you ever tested positive on a PCR test for COVID-19 after February 2022?” and answered “no” to the question: “Have you ever tested positive on a PCR test for COVID-19 before February 2022?”. The following note was also added for those in the later waves since there were cases in which positive results were determined by antigen tests or symptoms alone at the later waves: “Do not include a deemed positive result or a positive result based on antigen testing alone.”. The definitions of the early and late waves were based on the progression of the rate of infection in Japan as follows:^23^ there were several small waves of infections until September 2021, after which the number of infections remained minuscule^28^. Then, the number of infections increased rapidly in January 2022, reaching an unprecedented number in February 2022.

Participants who met the following criteria were excluded from the study: those who (a) selected the incorrect answer to the dummy question, (b) disclosed that they did not meet the inclusion criteria, (b) disclosed that they did not have PCR test, (c) provided inconsistent answers about their infection status, and (d) were outliers according to their answers in respect of demographic information.

### Measurement

Kessler Psychological Distress Scale (K6) was used as the outcome variable. K6 assesses the severity of psychological distress and consists of six items^29,30^. All items are evaluated on a four-point Likert scale, and a total score of five or more indicates that the individual has experienced at least mild psychological distress. A total score of 13 or more indicates that the individual has experienced severe psychological distress.

The experience of discrimination was defined and evaluated using information derived from previous studies as well as original questions developed by one of the authors based on their experiences after being infected with COVID-19^31^. First, the experience of being blamed by others for being infected were defined as those who answered that either of the following experiences applied to them: “Workers (bosses, colleagues, business partners, etc.) blamed me for being infected”, “Private parties (friends, neighbors, etc.) blamed me for being infected”. Second, the experience of some form of discrimination for being infected were defined as those who answered “yes” to the question: “Have you ever felt discriminated against or perceived discrimination for being infected with COVID-19”. Third, the experiences of participants and families being maligned were defined as those who answered “yes” to the question “Have you or your family ever been maligned by someone as a result of your infection status?”. The variables associated with both outcome variables and exposure variables were used as covariates: sex (male, female, others)^11,32^, age group (20–29, 30–39, 40–49, 50–59, 60–69, 70–79, 80–89, ≥ 90)^11,33–35^, level of education (junior high school, high school, vocational school or junior college, university, and postgraduate school)^11,36,37^, job status (employed and unemployed)^33,36,38^, Cohabitant status (living alone and living together)^11,35^, duration after infection^5,39^, hospitalization status at infection (hospitalized and not hospitalized)^3,40^. Sex was later converted to the binary variable by merging females and others. Level of education was divided into two categories: high school graduates or less. The variable of living alone was based on the answer to the question asking the participant the number of people they lived together with. Those who answered zero were categorized as living alone, while those who answered one or more were categorized as living together.

### Analyses

After calculating the mean and standard deviation (SD) for the continuous variables and the number and proportion for categorical variables, the differences between those infected in the early and later waves of COVID-19 were confirmed by χ^2^ tests and *t*-tests.

To confirm the incident risk of facing discrimination, Modified Poisson regression analyses were performed with three experiences of discrimination as criteria and timing of infection and covariates as predictors.

The difference in the degree of impact of the experiences of discrimination on psychological distress was confirmed with Modified Poisson regression analyses. A K6 score ≥ 5 was used as a criteria, and the timing of infection and experiences of discrimination as well as covariates were included in the model as predictors. Interaction effects between the timing of infection and the experiences of discrimination were also analyzed. E-values were also calculated for the significant association to confirm how large effect size of unmeasured cofounding variables would overturn the observed significant results. Modified Poisson regression analyses were additionally performed to confirm the relationships between the experiences of discrimination and K6 ≥ 5 for each timing of infection separately.

Supplemental analyses were also performed. An analysis of the interaction effects between timing of infection and experiences of discrimination and K6 ≥ 5 were performed excluding hospitalization from the covariates. This was done to avoid overadjustment for the timing of infection^41^, considering hospitalization was a intermediate variable from the timing of infection to the outcome. Analyses using K6 ≥ 13, instead of K6 ≥ 5, as a criterion were also performed.

Results with p-values less than 0.05 with two-tailed were considered significant. There were no missing values. Stata version 17 was used for analyses (College Station, TX: Stata Corp LLC).

### Ethics approval and consent to participate

This study was performed with the approval of the Ethics Committee of the NCNP in Japan (A2021-34) and following the Helsinki Declaration. All participants agreed to participate after obtaining informed consent through the online system^42^.

## Results

### Participants characteristics

**Figure 1:**
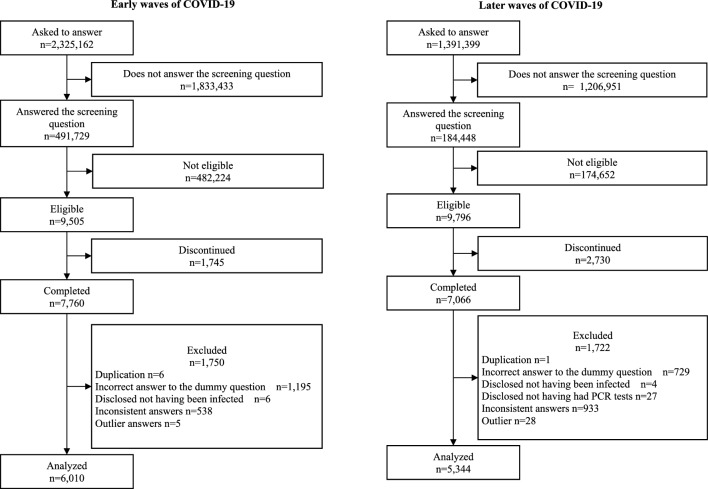
Flow chart.

Figure 1 shows the process of recruitment of participants as well as how the inclusion and exclusion criteria were applied. Out of 7760 possible participants who were infected the early waves of COVID-19, the data of 6010 individuals were analyzed, after 743 individuals’ questionnaires were excluded. Out of 7066 possible participants who were identified as being infected in the later COVID-19 waves, the data collected from 5344 individuals were analyzed after 1722 questionnaires were excluded.

Table 1 presents the demographic and other characteristics of the participants. The proportion of male participants was significantly larger in the early wave infected group than in the later wave infected group (57.19% vs 51.18%, p < 0.001). In respect of the age of the participants, most were between the age of 40 and 49 years old and approximately 90% of the participants were under the age of 60 years old in both the early wave infected and later wave infected groups, while the distribution was significantly different between the groups. The proportions of the following variables were also significantly different between the groups: the level of education (high school graduates or less, 22.56% vs 20.81%, p = 0.02), cohabitant status (living together, 80.65% vs 86.88%, p < 0.001), hospitalization (hospitalized or not, 24.43% vs 2.08%, p < 0.001), and duration after infection (4.99 months vs 2.62 months, p < 0.001). The proportion of participants who experienced psychological distress was significantly lower in the later wave infected group than in the early wave infected group (K6 ≥ 5, 39.52% vs 20.57%, p < 0.001; K6 ≥ 13, 9.12% vs 0.46%, p < 0.001).

**Table 1:** Participants’ characteristics.

	Total		Early waves of COVID-19 n = 6010		Later waves of COVID-19 n = 5344			*p*
	n	%	n	%	n	%		
Sex								
Male	6172	54.36	3437	57.19	2735	51.18	χ^2^ = 43.30	< 0.001
Female	5150	45.36	2553	42.48	2597	48.60		
Others	32	0.28	20	0.33	12	0.22		
Age group (in years)								
20–29	1815	15.99	1192	19.83	623	11.66	χ^2^ = 151.92	< 0.001
30–39	2928	25.79	1503	25.01	1425	26.67		
40–49	3191	28.10	1580	26.29	1611	30.15		
50–59	2294	20.20	1187	19.75	1107	20.71		
60–69	910	8.01	435	7.24	475	8.89		
70–79	198	1.74	101	1.68	97	1.82		
80–89	13	0.11	7	0.12	6	0.11		
≥ 90	5	0.04	5	0.08	0	0		
Level of education								
High school graduates or less	2468	21.74	1356	22.56	1112	20.81	χ^2^ = 5.12	0.02
Post-high school graduate	8886	78.26	4654	77.44	4232	79.19		
Job status								
Employed	9726	85.66	5151	85.71	4575	85.61	χ^2^ = 0.02	0.88
Unemployed	1628	14.34	859	14.29	769	14.39		
Cohabitation status								
Living together	9490	83.58	4847	80.65	4643	86.88	χ^2^ = 80.10	< 0.001
Living alone	1864	16.42	1163	19.35	701	13.12		
Hospitalization status at infection								
Hospitalized	1579	13.91	1468	24.43	111	2.08	χ^2^ = 1200.0	< 0.001
Not hospitalized	9775	86.09	4542	75.57	5233	97.92		
Duration after infection (months)	3.88	± 0.04	4.99	± 0.06	2.62	± 0.03	t = 35.82	< 0.001
K6 score	3.54	± 0.05	4.54	± 0.07	2.42	± 0.06	t = 23.43	< 0.001
≥ 5	3474	30.60	2375	39.52	1099	20.57	χ^2^ = 478.48	< 0.001
≥ 13	765	6.74	548	9.12	217	4.06	χ^2^ = 115.55	< 0.001
Experiences of discrimination								
Being blamed	641	5.65	548	9.12	93	1.74	χ^2^ = 289.06	< 0.001
Some form of discrimination	1820	16.03	1538	25.59	282	5.28	χ^2^ = 867.21	< 0.001
Participant or families being maligned	1206	10.62	1038	17.27	168	3.14	χ^2^ = 594.69	< 0.001

### The difference in the risk of experiencing discrimination between those infected in the early and late waves of COVID-19

As Table 1 shows, the experiences of being blamed, some form of discrimination, and participants and their families being maligned were significantly less in later wave infected group than in the early wave infected group, respectively (9.12% vs 1.74%, p < 0.001; 25.59% vs 5.28%, p < 0.001; 17.27% vs 3.14%, p < 0.001).

Incident risks of being blamed, experiencing some form of discrimination, and the participants or their families being maligned were significantly lower in the later wave infected group than in the early wave infected group after adjusted (Incident Relative Risk [IRR] = 0.26, 95% CI = 0.21–0.33, p < 0.001; IRR = 0.26, 95% CI = 0.23–0.29, p < 0.001; IRR = 0.25, 95% CI = 0.21–0.30, p < 0.001).

### The relationships between psychological distress, the experience of being blamed, and the timing of infection

The experience of being blamed and the infection timing of the early waves infected group were significantly associated with the risk of experiencing psychological distress (Table 2). A significant interaction between the experience of being blamed, the timing of infection and psychological distress was observed. E-value was 2.12. The incident risks of being blamed to psychological distress were 1.56 (95% CI = 1.45–1.68, p < 0.001) in the early infection COVID-19 group and 2.12 (95% CI = 1.69–2.65, p < 0.001) in the late COVID group.

**Table 2:** The relationships between psychological distress, being blamed, and the timing of infection.

	Crude				Adjusted				Interaction			
	IRR	95% CI		p	IRR	95% CI		p	IRR	95% CI		p
Timing of infection												
Early waves of COVID-19	1				1				1			
Later waves of COVID-19	0.55	0.52	0.58	< 0.001	0.58	0.55	0.62	< 0.001	0.58	0.54	0.62	< 0.001
Being blamed												
No	1				1				1			
Yes	1.74	1.62	1.86	< 0.001	1.62	1.51	1.74	< 0.001	1.56	1.45	1.68	< 0.001
Being blamed × the timing of the infection									1.39	1.10	1.75	0.007

The interaction effect of psychological distress was maintained when hospitalization status was excluded from the model (Appendix 1). When K6 ≥ 13 was used instead of K6 ≥ 5 as the criterion, a similar relationship was observed, but it was not significant (Appendix 2).

### The relationships between psychological distress, the experience of some form of discrimination, and the timing of infection

As is presented in Table 3, the experience of some form of discrimination was significantly associated with the risks of experiencing psychological distress independent from the timing of infection. The interaction effect with psychological distress was not significant. The incident risks of some form of discrimination to having psychological distress in the early COVID group and the late COVID group were 1.79 (95% CI = 1.69–1.90; p < 0.001) and 2.08 (95% CI = 1.79–2.42; p < 0.001), respectively.

**Table 3:** The relationships between psychological distress, some forms of discrimination, and the timing of infection.

	Crude				Adjusted				Interaction			
	IRR	95% CI		p	IRR	95% CI		p	IRR	95% CI		p
Timing of infection												
Early waves of COVID-19	1				1				1			
Later waves of COVID-19	0.61	0.57	0.65	< 0.001	0.64	1.73	1.94	< 0.001	0.62	0.58	0.67	< 0.001
Some forms of discrimination												
No	1				1				1			
Yes	1.91	1.81	2.02	< 0.001	1.84	1.73	1.94	< 0.001	1.79	1.69	1.90	< 0.001
Some forms of discrimination × the timing of the infection									1.17	0.997	1.37	0.055

A similar association was maintained when hospitalization was excluded from the model (Appendix 1). When K6 ≥ 13 was used instead of K6 ≥ 5 as the criterion, the opposite association was observed in the interaction effect to experiencing psychological distress, but it was not significant (Appendix 2).

### The relationships between psychological distress, the experiences of participants and their families being maligned, and the timing of infection

The experience of participants and their families being maligned was significantly associated with the risk of experiencing psychological distress independent of the timing of infection, although the interaction effect of experiencing psychological distress was not significant (Table 4). The incident risks of participants and their families being maligned to experiencing psychological distress in the early and late waves of COVID-19 was 1.81 (95% CI = 1.70–1.93, p < 0.001) and 2.10 (95% CI = 1.75–2.51; p < 0.001).

**Table 4:** The relationships between psychological distress, being maligned, and the timing of infection.

	Crude				Adjusted				Interaction			
	IRR	95% CI		P	IRR	95% CI		p	IRR	95% CI		P
The timing of infetion												
Early waves of COVID-19	1				1				1			
Later waves of COVID-19	0.59	0.55	0.63	< 0.001	0.61	0.57	0.65	< 0.001	0.60	0.56	0.65	< 0.001
Being maligned												
No	1				1				1			
Yes	1.93	1.83	2.05	< 0.001	1.86	1.75	1.97	< 0.001	1.82	1.71	1.94	< 0.001
Participant or families being maligned × The timing of infection									1.17	0.97	1.41	0.10

These relationships were maintained when hospitalization was excluded from the model (Appendix 1) and when K6 ≥ 13 was used as the criterion instead of K6 ≥ 5 (Appendix 2).

## Discussion

This cross-sectional study investigated the difference in the risks of facing discrimination based on COVID-19 infection status and risks of experiencing psychological distress between those who were infected in the early and later COVID waves. The risks of facing discrimination due to COVID-19 infection status, such as the experiences of being blamed, some form of discrimination, and participants and families being maligned were found to be lower in those who were infected in the later waves of COVID-19 than those in the early waves. On the other hand, the risks of facing discrimination, especially being blamed for one’s own COVID-19 infection, on psychological distress, were indicated to be more serious among those who were infected in the later waves than among those infected in the early waves.

The risks of perceiving discrimination were lower in those who had been infected in the later waves than those who had been in the early waves. This result was consistent with a previous study indicating that discrimination frequency was lower in 2021 than it was in 2020^10^. Although inconsistent with the results of a study conducted on the general population claiming that discrimination frequency had not decreased after a month duration^22^. Based on the difference in results, it can be inferred that a longer period between the first and second points of measurement would result in a noticeable reduction. The reduction of discrimination experienced from the time of the early waves to the later waves of COVID-19 may be accounted for by the accumulation of public knowledge relating to COVID-19 over time. The more knowledge the public gains about COVID-19, the weaker the stigmatic climate should be^19^ and the amount of knowledge about COVID-19 held by the public increases as time passes^21^.

The risks of being blamed for one’s own COVID-19 infection to experiencing psychological distress were indicated to be higher in those who were infected in the later waves than those infected in the earlier wave, although the experiences of some forms of discrimination and participants being maligned for having been infected with COVID-19 were not significant. A previous study reported that the discrimination stress was comparable between early COVID-19 infection and late infection^10^. The degree of impact on psychological distress in early waves and later waves may vary depending on the types of discrimination. It is suggested that the reason the risk of facing discrimination to psychological distress was stronger in the later wave infection group than in the early wave infection group is due to difficulty in the development of an identity as a member of the COVID-19 survivors’ group that has faced discrimination in the late wave infection group. This refers to a concept known as the rejection-identification model^24^ and group identification, recognizing themselves as a member of a specific handicapped group as distinct from powerful majority^23^. The rejection-identification model presents that the group identification is a buffering factor between discrimination and psychological distress^24–26^. Group identification is generally developed by gathering many people who perceived discrimination for similar handicaps^24–26^. Group identification is developed more actively in societies with stronger discriminatory climates than in societies with weaker discriminatory climates^27^. The COVID-19 survivors facing discrimination were found to be fewer in late COVID in this study. This may result in difficulty in gathering to develop a group identification as a member of discriminated COVID-19 survivors. Therefore, those in the late wave were considered to be difficult in buffering the psychological distress caused by discrimination with group identification as discriminated COVID-19 survivors. Furthermore, the experience of being blamed had more of an effect on psychological distress than the experience of some forms of discrimination and being maligned. The difference in the strength of the association between each experience and psychological distress could be caused by whether each experience includes indirect discrimination or not. This is because, unlike direct discrimination, the effects of indirect discrimination on psychological distress have not been shown to be buffered by group identification^25^. The experience of being blamed refers to direct discrimination only. The experience of some forms of discrimination and being maligned were comprehensive experiences of discrimination, including indirect discrimination, such as discrimination against their families or social climates. Thus, the degree of relationships between the latter two and psychological distress might not be influenced by the strength of discriminatory climate in the society during the COVID-19 pandemic.

The risk of experiencing psychological distress was lower in the later waves infected group than in the early waves infected group. This finding was inconsistent with the studies conducted in Norway and the United States of America, which showed comparable risks between early and later waves^9,10^. This difference in the prognosis after COVID-19 is suggested to be induced by the difference in environments by countries, such as the timing of the pandemic and policy considerations^43^, as well as cultural differences during COVID-19. The discrepancy of the number of infected people per million between those who were infected during the early waves and those who were infected during the later waves was larger in Japan than in other countries^44^. Thus, the shift from the perception that infection is specific to the perception that anyone can be infected may be more pronounced than in other countries. Furthermore, considering more responsive to the social condition due to collectivism cultures in Asia than in Western countries^45,46^, COVID-19 survivors in Japan may be more responsive to social change related to COVID-19 such as the number of infected people and policy consideration than in Norway and USA. This may drive remarkable change in psychological distress among those in Japan.

To improve psychological distress caused by discrimination among COVID-19 survivors in the late waves, psychological interventions may be needed. For example, the peer-support group may be effective based the studies that show the improvement of psychological distress and self-stigma in several conditions^47,48^. The peer support group might also play a role in developing group identification, as it allows discriminated COVID-19 survivors to gather and communicate with each other despite there are few discriminated COVID-19 survivors. Studies examining the effectiveness of peer support groups for discriminated COVID-19 survivors should be conducted in the future.

Our study had some limitations. First, most of participants were 20 years old or older, but were under the age of 60 years old. Therefore, our findings cannot be applied to all age groups. Second, causal relationships remain unclear due to the cross-sectional nature of the study design. Thus, we submit that a longitudinal study should be conducted. Third, there might be potential confounders associated with psychological distress, although the potential confounders must have an RR of at least 2.12 for the outcome, independent of all measured covariates, for the main results of the study to be overturned. Especially, the vaccination status was not adjusted in this study. The vaccination status may potentially influence the results based on the studies indicating the effect of the vaccination on PASC reduction^49^ and the association between vacctination stat us and discrimination^50^. Forth, the reliability and validity of the items relating to experiences of discrimination were not confirmed, since there is no validated scale concerning discrimination specific to COVID-19 infection.

## Conclusion

Our results revealed that the risk of perceiving discrimination against COVID-19 infection was lower among COVID-19 survivors who were infected in the later waves than in the earlier waves, while the risk of discrimination for COVID-19 to psychological distress was more serious in those who were infected in the later waves than in the earlier waves. Therefore, we submit that a counterplan focusing on those survivors who have experienced discrimination in relation to their COVID-19 infection status may become necessary in the future rather than only focusing on society as a whole to reduce the discriminatory climate.

### Supplementary Information

Supplementary Information 1.

Supplementary Information 2.

## Data Availability

The datasets generated during and/or analyzed during the current study are not publicly available but are available from the corresponding author on reasonable request.
