# Supplementary material for: Difference in the risk of discrimination on psychological distress experienced by early wave infected and late wave infected COVID-19 survivors in Japan
Source: Sci Rep. 2023 Aug 12;13:13139. doi: 10.1038/s41598-023-40345-9 (PMC10423270; doi:10.1038/s41598-023-40345-9)
Supplement: Supplementary file 1 — Supplementary Information 1. [file 41598_2023_40345_MOESM1_ESM.docx]

**Appendix 1. The relationships between psychological distress, the experiences of discrimination, and the timing of infection after the hospitalization excluded**

|  | **Being blamed** | | | |
| --- | --- | --- | --- | --- |
|  | **IRR** | **95% CI** | | **p** |
| The timing of infection |  |  |  |  |
| Early waves of COVID-19 | 1 |  |  |  |
| Later waves of COVID-19 | 0.54 | 0.50 | 0.57 | <0.001 |
| Being blamed |  |  |  |  |
| No | 1 |  |  |  |
| Yes | 1.63 | 1.51 | 1.76 | <0.001 |
| Being blamed  ×  The timing of infection | 1.34 | 1.06 | 1.76 | <0.001 |
| The timing of infection |  |  |  |  |
| Early waves of COVID-19 | 1 |  |  |  |
| Later waves of COVID-19 | 0.60 | 0.56 | 0.64 | <0.001 |
| Some forms of discrimination |  |  |  |  |
| No | 1 |  |  |  |
| Yes | 1.87 | 1.76 | 1.98 | <0.001 |
| Some forms of discrimination  ×  The timing of infection | 1.13 | 0.96 | 1.32 | 0.14 |
| The timing of infection |  |  |  |  |
| Early waves of COVID-19 | 1 |  |  |  |
| Later waves of COVID-19 | 1.13 | 0.93 | 1.35 | <0.001 |
| Being maligned |  |  |  |  |
| No | 1 |  |  |  |
| Yes | 1.9 | 1.80 | 2.03 | <0.001 |
| Participants or families being maligned  ×  The timing of infection | 1.13 | 1.25 | 0.21 | 0.21 |
