# Supplementary material for: Difference in the risk of discrimination on psychological distress experienced by early wave infected and late wave infected COVID-19 survivors in Japan
Source: Sci Rep. 2023 Aug 12;13:13139. doi: 10.1038/s41598-023-40345-9 (PMC10423270; doi:10.1038/s41598-023-40345-9)
Supplement: Supplementary file 2 — Supplementary Information 2. [file 41598_2023_40345_MOESM2_ESM.docx]

**Appendix 2. The relationships between severe psychological distress, the experiences of discrimination, and the timing of infection**

|  | **Being blamed** | | | |
| --- | --- | --- | --- | --- |
|  | **IRR** | **95% CI** | | **p** |
| The timing of infection |  |  |  |  |
| Early waves of COVID-19 | 1 |  |  |  |
| Later waves of COVID-19 | 0.56 | 0.47 | 0.67 | <0.001 |
| Being blamed |  |  |  |  |
| No | 1 |  |  |  |
| Yes | 2.44 | 2.02 | 2.93 | <0.001 |
| Being blamed  ×  The timing of infection | 1.58 | 0.94 | 2.64 | 0.08 |
| The timing of infection |  |  |  |  |
| Early waves of COVID-19 | 1 |  |  |  |
| Later waves of COVID-19 | 0.71 | 0.59 | 0.86 | <0.001 |
| Some forms of discrimination |  |  |  |  |
| No | 1 |  |  |  |
| Yes | 3.04 | 2.58 | 3.56 | <0.001 |
| Some forms of discrimination  ×  The timing of infection | 0.91 | 0.61 | 1.37 | 0.66 |
| The timing of infection |  |  |  |  |
| Early waves of COVID-19 | 1 |  |  | <0.001 |
| Later waves of COVID-19 | 0.62 | 0.52 | 0.74 |  |
| Being maligned |  |  |  |  |
| No | 1 |  |  | <0.001 |
| Yes | 2.69 | 2.78 | 3.17 |  |
| Participants or families being maligned  ×  The timing of infection | 1.04 |  |  | 0.88 |
